# Practical Observations on Catarrhal Ophthalmia, and on the Contagious Ophthalmia to Which It Gives Rise; with Cases

**Published:** 1826-10

**Authors:** Wm. Mackenzie

**Affiliations:** Andersonian Professor of Anatomy and Surgery; and one of the Surgeons to the Glasgow Eye Infirmary.


### OPHTHALMIA.

*Practical Observations on Catarrhal Ophthalmia, and on the contagious Ophthalmia to which it gives rise; with Cases.* By WM. MACKENZIE, Andersonian Professor of Anatomy and Surgery; and one of the Surgeons to the Glasgow Eye Infirmary.

THE ophthalmiæ which are most frequently excited, in adults, by atmospheric influences, are these three—the *catarrhal,* the *rheumatic,* and the *catarrho-rheumatic.*

Although the distinction of these three diseases must be familiar to all who have studied in the German schools, or by reading made themselves acquainted with the works of the German oculists, I believe the distinction is but rarely attended to in this country, even by oculists, and still less by general practitioners. Some, indeed, have attempted to throw ridicule upon any thing like a distinction of genera and species in the various diseases of the eye. An ophthalmia, a cataract, and an amaurosis, are sufficient nosological distinctions for their general and superficial views. At the same time, I am certain, not only of the reality and truth of the distinctions to which I have already referred, and of many others among the inflammatory diseases of the eye, not yet generally received,—but I am convinced also, after considerable experience in the treatment of these diseases, that such distinctions are absolutely necessary to be known, if we mean to treat the various kinds of ophthalmia with success. The catarrhal, rheumatic, and catarrho-rheumatic ophthalmiæ, for example, cannot be treated successfully by one common method. Treated without discrimination, they but too often prove destructive of vision; whereas, by appropriate treatment, they are in general easily managed. The appropriate treatment of the rheumatic ophthalmia, however, is not at all adapted to the catarrhal; while the remedies which in a few days subdued the catarrhal, would only exasperate the rheumatic.

The first of the three diseases above named is an affection of that muco-cutaneous membrane which lines the eyelids, and covers the anterior third of the eyeball, the *conjunctiva;* a continuation at once of skin, liable to eruptive or cutaneous diseases; and a continuation of the vast mucous membrane which lines the respiratory and alimentary passages, liable therefore to puro-mucous or blenorrhœal diseases. The second is an affection of the fibrous *sclerotica*, and surrounding fibrous membranes. In the third, both the *conjunctiva* and the *sclerotica* are affected, and the symptoms of the catarrhal are united to those of the rheumatic ophthalmia.

I have been in the habit of speaking of inflammation of the conjunctiva, under the name of *conjunctivitis;* and of those inflammations of that tunic which affect it as a mucous membrane, under the generic name of *conjunctivitis puro-mucosa.* Of this genus, the following are the species—

1. Conjunctivitis puro-mucosa atmospherica; Catarrhal ophthalmia.
2. — — contagiosa; Egyptian.
3. — — leucorrhoica; Ophthal.-neonatorum.
4. — — gonorrhoica.

The inflammation in the Catarrhal Ophthalmia, which is by far the most common disease of the eye in adults, is almost entirely confined to the conjunctiva and Meibomian follicles. The mucous secretion of the membrane is increased in quantity, and occasionally becomes opake, thick, and puriform; but in many cases it remains transparent, and, from its superabundant quantity, merely renders the eyelids more than usually moist and slippery; while the Meibomian secretion, also increased in quantity and changed by disease, concretes on the edges of the eyelids and amongst the eyelashes, and binds them together during the night.

In mild cases, the redness is chiefly in the conjunctiva lining the eyelids. On the white of the eye, the vessels are arranged in a net-work, and can be moved in every direction, by pressing the eyelid against the eyeball with the finger, showing that they reside in the conjunctiva. In severe cases, chemosis takes place, even to a great extent; so much so, that if general treatment only be employed, such as blood-letting and purging, while local means are neglected, the cornea may lose its vitality, burst, and slough, from the mere pressure to which the eye is subjected, and thus vision be destroyed. I attribute the destruction of the cornea in severe cases of catarrhal ophthalmia, as also in the contagious or Egyptian ophthalmia, and in the ophthalmia of new-born children, not so much to a vital as to a mechanical cause,—not to excessive inflammatory action in the cornea itself, but rather to the pressure caused by the enormously distended conjunction of the eyelids and eyeball. Other causes, no doubt, concur, in the puro-mucous inflammations of the conjunctiva, to produce opacities of the cornea, detachment of its conjunctival covering, and ulceration; and, in particular, the maceration of the cornea in a flood of purulent fluid, not sedulously removed by injections. But the destruction of the cornea by sloughing, I am disposed to refer more to pressure and mechanical death, than to violent inflammatory action.

In the catarrhal ophthalmia, the patient uniformly complains of a feeling of sand in the eye, or of broken glass rolling under the upper eyelid; a sensation which never attends the pure rheumatic ophthalmia, and may therefore be regarded as strikingly *diagnostic*. Moreover, in the catarrhal ophthalmia, the patient is usually free from headache; whereas, in the rheumatic, one of the most remarkable symptoms, and by far the most distressing, is circumorbital pain, severely aggravated during the night. When headache does attend catarrhal ophthalmia, it is seated across the forehead, and is felt most in the morning.

So distressing, even at the beginning of an attack of catarrhal ophthalmia, is the sensation as if sand or some other foreign body were under the upper eyelid, that I have repeatedly been requested to visit patients, in whom this disease was commencing, who supposed that some particle of dust had actually got into that situation; and in one instance I was called to visit a medical gentleman, who was so convinced, from the feeling which he experienced, that this was the case, that he had made various attempts, with his dressing probe, to free himself from the imaginary offending substance.

Atmospheric changes, and especially exposure to cold and wet, are the exciting *causes* of this disease. Night watching, and exposure to the night-air in a state of intoxication, are frequently the occasions which give rise to catarrhal ophthalmia. Wet feet is a cause which some of my patients have particularly mentioned. An individual who has once laboured under this disease, is more likely to be attacked again: one of my patients had three attacks between May and January.

In many instances, the catarrhal ophthalmia has been known suddenly to attack a great number of persons, who happened to be exposed to the same general exciting causes. Assalini relates, for instance, that, in May 1792, several battalions of the Duke of Modena's troops arrived at Reggio, in order to quell some riots. These troops passed the first night after their arrival under the spacious porticoes of a convent looking to the north, in the lowest part of the town, and near the trenches of the citadel. Many of these soldiers contracted a violent catarrhal ophthalmia, which was attributed to the dust of the straw on which they had slept, and not to the moist and cold air of the place, which no doubt was the true cause, and which was so much the more likely to prove hurtful, as these men had been accustomed to close and comfortable quarters.*

The catarrhal ophthalmia has been known to spread itself still more extensively, attacking a great proportion of the inhabitants of a town or district, so as to obtain the name of *epidemic ophthalmia*. In 1778, it attacked the whole neighbourhood about Newbury in Berkshire; and, in the same year, it prevailed in several of the English camps, where it was known by the name of the *ocular disease*. In 1806, an epidemic ophthalmia of this kind prevailed in Paris, and was in many cases attended by an affection of the mucous membrane of the air-passages; a complication which I have repeatedly observed in the sporadic cases of this country.

* Manuale di Chirurgia, pa[illegible] 2, p. 117.

The same disease prevailed in 1808, at Vicenza in Italy. It has been mentioned by some authors, that this disease is more common in summer and autumn. In this town and neighbourhood, it is common at all seasons.

If the catarrhal ophthalmia, or atmospheric puro-mucous conjunctivitis, be neglected, or treated only with general remedies, or with improper local ones, it will continue for many weeks, and become the cause of much febrile excitement and constitutional illness, as well as local distress. Amongst other bad effects of neglect, the conjunctiva, particularly of the upper eyelid, is apt to become sarcomatous and rough, and, by rubbing in this state against the cornea, brings on vascular nebula, and even dense white opacity, especially of the upper half of the cornea. The discharge from the conjunctiva is more apt, also, under neglect or improper treatment, to become puriform; and, being conveyed from the eyes of the patient to those of others, by actual contact, or by the use of towels and the like in common, will excite a conjunctivitis still more severe, more distinctly puriform, and more dangerous in its effects on the transparent parts of the eye. This, at least, is the conclusion at which I have arrived, from the observation of many instances, in which, as far as it was possible to come to the facts, this disease having arisen in one member of a family from atmospheric exposure, several others of the family have become affected without any such exposure, that could be ascertained; and while, in the first affected, the disease was comparatively moderate and scarcely puriform, in the latter the symptoms were more violent, and the discharge was thick, abundant, and opake.

I think it probable, that the ophthalmia also which attacked the British and French armies in Egypt was an atmospheric puro-mucous conjunctivitis, but that it afterwards degenerated into a contagious, perhaps infectious, disease,—propagated, that is to say, by actual contact of the discharge, and perhaps by miasmata from the discharge floating through the air. Nor is this idea inconsistent with what is generally admitted regarding contagious and infectious diseases. If we admit such a thing as contagion or infection at all, we must also admit, I should apprehend, that diseases, originally excited by external influences, were propagated only, in the second and succeeding instances, by their contagious or infectious power.

I know of no experiments in which the discharge from an eye affected with puro-mucous conjunctivitis, arising from atmospheric influence, has been applied to a sound eye. Dr.

Guillié's experiments, indeed, may have been performed with matter of this description. He took the puriform mucus from the eyelids of some children affected with puro-mucous conjunctivitis, in the Hospital for Sick Children at Paris, and introduced it under the eyelids of four blind children belonging to the Institution for the Blind. These children were amaurotic, but the external surface of their eyes was healthy and entire. In all four a regular puro-mucous conjunctivitis was produced.*

The following I regard as a striking, and indeed fearful, instance of catarrhal ophthalmia, or puro-mucous conjunctivitis, excited by atmospheric influence, spreading by contagion.

The French slave-ship Rôdeur, Captain B—, of two hundred tons burden, left Hâvre on the 24th January, 1819, for the coast of Africa, reached her destination on the 14th March, and cast anchor off Bonny, in the river Calabar. The crew, of twenty-two men, enjoyed good health the whole voyage, and during their stay at Bonny, till the 6th of April. No trace of ophthalmia had been observed among the inhabitants of the coast, and it was not till fifteen days after the Rôdeur had put to sea, and was nearly on the equator, that the first symptoms of this frightful disease were perceived.

It was observed that the negroes, who were 160 in number, and crowded together in the hold and between decks, had contracted a considerable redness of the eyes, which spread with rapidity from one to another. At first, however, the crew paid no great attention to this appearance, imagining that it was occasioned merely by want of fresh air in the hold, and by the scarcity of water; for they already limited the allowance of water to eight ounces a-day, and some time after they could allow only half a glass a-day. It was thought sufficient to make use of an eye-water made from an infusion of elder-flowers, and (following the advice of one Maignan, who acted as ship-surgeon,) to bring up the negroes in turns upon deck. This salutary measure, however, they were obliged to abandon; for the poor Africans, torn from their native home, and heart-wrung by the horrors of their situation, as well as by the recollections of their lost freedom, embracing each other, threw themselves into the sea.

The disease, which had spread amongst the negroes in a frightful and rapid manner, did not tarry to become contagious for all, and to threaten even the crew. The first man of the crew attacked was a sailor who slept under deck,

* Bibliothèque Ophthalmologique, tome i. p. 81.

close to the grated partition which communicated with the hold. Next day, a lad was affected with the ophthalmia; and, in the course of the next three days, the captain, and almost all the crew, were seized.

In the morning, on awakening, the patients experienced a slight prickling and itching in the edges of the eyelids, which became red and swoln. Next day, the swelling of the eye-lids was increased, and attended with sharp pain; in order to lessen which, they applied to the eyes poultices of rice, as hot as they could bear them. On the third day of the disease, a discharge of yellowish matter took place, rather thin at first, but which afterwards became viscid and greenish; and was so abundant, that the patients had only to open their eyes every quarter of an hour, when the matter fell in drops. From the commencement of the disease, there were considerable intolerance of light and discharge of tears. When the rice failed, boiled vermicelli was used for poultices. On the fifth day, blisters were applied to the nape of the neck of some of the patients; but, as the cantharides were soon exhausted, they endeavoured to supply their place by the use of pediluvia containing mustard, and by exposing the swoln eyelids to the steam of hot water.

Far from diminishing under this treatment, the pain increased from day to day, as well as the number of those who lost their sight; so that the crew, besides fearing a revolt amongst the negroes, were struck with terror lest they should not be able to manage the vessel till they should reach the Carribbee Islands. One sailor only had escaped the contagion, and upon him their whole hopes depended. The Rôdeur had already fallen in with a Spanish ship, the Leon, whose whole crew were so affected with the same disease, that they could no longer manage their ship, but begged the aid of the Rôdeur, already almost as helpless as themselves. The seamen of the Rôdeur, however, could not abandon their own ship, on account of the negroes; nor had they room to receive the crew of the Leon. The difficulty of nursing so many patients in so narrow a space, and the want of fresh provisions and of medicines, made the survivors envious of those who died; a fate which seemed to be fast coming upon all, and the thought of which caused general consternation.

Some of the sailors made use of brandy, which they dropped between their eyelids, and from which they experienced some relief; which might have suggested to the surgeon the propriety of a local stimulating treatment.

On the twelfth day, the sailors who had experienced some relief came upon deck, to relieve the others. Some were thrice attacked with the disease.

The tumefaction of the eyelids having subsided, some phlyctenulæ were observed on the conjunctiva of the eyeball. These the surgeon had the imprudence to open; a step which proved hurtful in his own case, for he remained blind, without any possibility of recovering his sight.

On reaching Guadaloupe, on the 21st June, the crew was in a deplorable state; but, very soon after, from the use of fresh provisions, and by simple lotions of spring-water and lemon-juice, recommended by a negress, they became sensibly better. Three days after coming ashore, the only man who during the voyage had escaped the contagion, was in his turn seized with the same symptoms; the ophthalmia running its course as it had done on-board ship.

Of the negroes, thirty-nine remained blind, twelve lost each one eye, and fourteen had specks, more or less considerable, of the cornea.

Of the crew, twelve men lost their sight; one of these was the surgeon. Five lost each one eye; and amongst these was the captain. Four had considerable specks, and adhering of the iris to the cornea.*

The atmospheric puro-mucous conjunctivitis, or catarrhal ophthalmia, yields readily, in general, to a very simple treatment, chiefly of a local and stimulating kind. I was first struck with the truth of this fact, in the successful treatment of this disease by Professor Beer, at Vienna, in 1817. I have since been greatly confirmed in the opinion that general remedies are inferior in importance to local ones, in this disease; that violent general remedies are absurd, and worse than useless; and that a local stimulant treatment may almost entirely be relied on, from the cases detailed in the excellent Report by Mr. Melin, published in the London Medical and Physical Journal for September 1824, and from the results of my own practice, both in private and at the Eye Infirmary.

1. I very rarely find it necessary to take away blood in catarrhal ophthalmia, either from a vein or by leeches. When there is more than usual constitutional irritation, the taking away of from twelve to twenty ounces of blood from the arm, will no doubt prove useful; but this will rarely be necessary, if the disease has not been neglected for a number of days, or mistreated.

2. Scarification of the conjunctiva of the eyelids is necessary only in cases in which there is some degree of chemosis, and a distinctly puriform discharge. In such cases it proves a valuable means of cure. One or two deep incisions being

* Bibliothèque Ophthalmologique, tome i. p. 74.

The thieves had no right to be flaying.

made along the inner surface of the upper or lower eyelid, a very considerable discharge of blood will immediately take place; and, if the eyelid be properly managed, blood will continue to flow for a considerable time. For this purpose, the eyelid ought neither to be held everted till the bleeding ceases, nor allowed to fall back into continued contact with the eyeball, in either of which cases it will soon cease; but the eyelid ought to be alternately everted and permitted to return to its natural position, by which means the divided vessels are refilled, and thus a continual flow of blood is produced.

3. A brisk dose of calomel and jalap may be ordered, with occasional doses of neutral salts.

4. Determining to the skin is also useful; which may be done by the warm pediluvium at bed-time, and by small doses of Spiritus Mindereri, or of any other mild diaphoretic, in combination with diluent drinks.

5. In severe cases, a blister to the back of the neck will be found useful, or blisters behind the ears.

6. Even weak solutions of acetate of lead, or of sulphate of zinc, are prejudicial in this disease, aggravating the sensation as if sand were in the eye, increasing the redness, and leading to opacities and ulcers of the cornea.

7. On the contrary, the feeling of sand is uniformly relieved, and the inflammation abated, by the use of the solution of nitrate of silver. The solutions which I employ contains from two to four grains of the nitrate in one ounce of distilled water. A large drop is to be applied to the eye once a-day, by means of a camel-hair pencil. The instant that it touches the eye, the salt is decomposed, and the silver precipitated over the conjunctiva in the state of muriate. I have sometimes alarmed other practitioners, by proposing to drop upon the surface of an eye highly vascular, affected with a feeling as if broken pieces of glass were rolling under the eyelids, and evidently secreting purulent matter, a solution of lunar caustic; and I have been not a little amused and pleased at their surprise, when next day they have found all the symptoms much abated by the use of this application.

8. As a collyrium, I am in the habit of using a solution of one grain of corrosive sublimate in eight ounces of water. This being made milk-warm, is used thrice a-day for fomenting the eyelids, by means of a linen rag. In mild cases, a few drops are thus allowed to flow in upon the eye; but, in severe cases, in which the discharge is copious and puriform, this collyrium must be injected over the whole surface of the conjunctiva, and especially into the upper fold of that membrane, by means of a syringe; so that the whole morbid secretion is removed, and the diseased membrane immediately touched by the solution.

9. At bed-time, about the size of a large pin-head of red precipitate ointment, melted on the end of the finger, is to be smeared along the edges of the eyelids. This ointment is prepared by levigating twelve grains of red precipitate till they become an orange-coloured impalpable powder, to which one ounce of fresh butter is to be added. I have occasionally seen this ointment prepared so carelessly, that crystalline scales of red precipitate were evident in it to the naked eye. The red precipitate ought to be carefully levigated till it lose the red colour, and become orange. Added to the quantity of unctuous substance above mentioned, it forms a golden-coloured ointment, which keeps for a great length of time, and is by far the best of all eye-salves.

10. The inside of the upper eyelid ought daily to be inspected. If there is any tendency to a rough and sarcomatous state of the conjunctiva, it ought to be touched with the solid sulphate of copper.

I have treated many cases of catarrhal ophthalmia according to this plan, and with uniform success. In no case, in which the above simple remedies were had recourse to previously to ulcer or opacity of the cornea, did any ulcer or opacity ever occur; nor did the symptoms ever fail speedily to subside. On the other hand, I have repeatedly had occasion to see cases of this disease, which had been much aggravated by trusting altogether to general treatment, and especially to bleeding; or by the use of acetate of lead, or sulphate of zinc, as local applications. I have been led to attribute to these salts the detachment of the conjunctival layer of the cornea, and at any rate the formation of an opake cicatrix of the cornea; whereas, such superficial ulcerations, treated with the solution of nitrate of silver, have uniformly healed without opacity.

An analogous mode of treatment is to be followed in the different contagious species of puro-mucous conjunctivitis. They are all, however, much more severe and more dangerous diseases than the catarrhal. But any further remarks upon them, I must leave till some future opportunity. In the mean time, I shall add a few cases illustrating the progress of the cure of the catarrhal ophthalmia, and of the contagious ophthalmia to which it gives rise, under the plan of treatment which I have recommended. The contagious cases yield readily, in general, to the same means as the catarrhal; only it must be allowed that depletion and rest are more necessary.

CASES.

Case I.—June 23d, 1824.—Margaret Forsyth, age thirty-four. Scattered redness of both conjunctivæ, with itchiness and epiphora; eyelids glued in the morning. These symptoms have continued for eight days.

Pulv. Jalap. gr. xv.; Subm. Hydr. gr. vj. M. capiat q. p.—Mur. Hydr. gr. j.; Aquæ ℥ vj. Solve pro collyrio, ter indies utendo.

25th.—Redness and other symptoms entirely gone.

Case II.—November 1st, 1824.—Mary Bell, aged four and a half years. Puro-mucous conjunctivitis of eight days' standing; apparently from contagion. The discharge copious and opake.

Gtt. Sol. Nitr. Argent.—Collyr. Mur. Hydrarg.

3d.—Puriform discharge diminished.

Ung. Præcip. Rub. marginibus palpebræ o. n.—Cont. Solut. et Collyrium.

5th.—Puriform discharge much less.

12th.—Still improves.

Continuantur Unguentum et Collyrium.

December 1st.—Dismissed cured.

Case III.—November 10th, 1824.—Elizabeth Miller, age nine months. Catarrhal ophthalmia of six days' standing: left upper eyelid very much swelled.

Pulv. Jalapæ gr. vj.; Subm. Hydr. gr. ij. M. capiat q. p.—Gtt. Sol. Nitr. Argent.—Cataplasma super oculum sinistrum.

12th.—Swelling of the eyelid somewhat abated, and discharge diminished. Bowels confined.

Rep. Pulv. purgans.—Collyr. Mur. Hydr.

19th.—Symptoms much abated.

Cont. Collyrium.

December 1st.—Dismissed cured.

Case IV.—December 27th, 1824.—Agnes Munro, age thirty-five. Catarrhal ophthalmia of the right eye, which is affected with considerable pain.

Gtt. Solut. Nitrat. Argent.—Ung. Præcipit. Rub. o. n.—Collyr. Muriat. Hydrargyri.

29th.—Eye rather easier. Headache through the night.

Capiat Pulv. Ipecac. et Opii ℈j. h. s.

January 7th, 1825.—All but well. 10th.—Dismissed cured.

Case V.—January 12th, 1825.—Walter Tennant, age thirty-two. Catarrhal ophthalmia of both eyes, chiefly affecting the right, but shifting from eye to eye for the last six months.

Gtt. Solut. Nitr. Argent.—Ung. Præcip. Rub. o. n.

14th.—Eyes less painful, but the puriform discharge more copious; right eye less red.

Collyrium Muriat. Hydrarg.

17th.—Much improved.

Case VI.—May 20th, 1825.—Margaret Muir, age twenty-two months. Considerable inflammation of the left conjunctiva, with a discharge of puriform matter. Symptoms began a fortnight ago.

Gtt. Solut. Nitr. Argent.—Ung. Præcip. Rub. o. n.—Vesicat. pone aurem sinistram.

23d.—Puriform discharge considerably less.

Collyrium Muriat. Hydrarg.

30th.—Discharge entirely ceased.

Cont. Collyrium.

June 6th.—Dismissed cured.

Case VII.—June 8th, 1825.—Grace Eccles, age two and a half years. Severe inflammation of the palpebral conjunctiva, with chemosis, and puriform discharge, attended with the eruption of measles.

Gtt. Solut. Nitrat. Argent.—Collyr. Mur. Hydr.—Ung. Præcip. Rub. o. n.

10th.—Swelling of the eyelids fallen, but puriform discharge more abundant.

Injiciatur supra oculos Solutio Sulphat. Cupri.—Infusi Fol. Sennæ ℨj.—Cont. Collyrium et Unguentum.

13th.—Much improved.

Repet. Solutio Nitr. Argent.—Cont. Collyrium.

15th.—Pulv. Rhei gr. viij.

July 1st.—Repet. Pulv. Rhei.

25th.—Dismissed cured.

Case VIII.—July 15th, 1825.—Mary Ann M'Intyre, age three. Conjunctivitis purulenta, of a fortnight's standing. Eyelids so much swollen that the cornea cannot be seen.

Scarif. facies interna palpebrarum.—Unguentum Præcip. Rub. o. n.—Vesicatoria pone aures.

18th.—Injiciatur Sol. Sulphat. Cupri supra oculos.—Collyr. Muriat. Hydrarg.

22d.—Discharge much diminished, and swelling of conjunctiva fallen.

Case IX.—November 18th, 1825.—Elizabeth Sutherland, age fifty-one. Conjunctivitis catarrhalis of the left eye, of a fortnight's standing.

Subm. Hydrarg. gr. v.; Pulv. Jalapæ gr. xv. M. capiat q. p.—Collyrium Muriat. Hydrarg.

21st.—Scarif. facies interna palpebr. infer. sinist.—Ung. Præcip. Rubr. o. n.

23d.—Vesicatoria pone aurem sinistram.

30th.—Repet. scarificatio.

December 21st.—Eyes almost perfectly well.

Cont. Unguentum et Collyrium.

January 9th, 1826.—Dismissed cured.

Case X.—December 28th, 1825.—Maria Duffie, age two and a half years. Lost the sight of the left eye when a month old. Conjunctivitis catarrhalis, of a month's standing, consequent to measles.

Gtt. Sol. Nitr Argent.—Collyr. Muriat. Hydr.—Ung. Præcip. Rubr. o. n.

30th.—Symptoms much abated.

Cont. medicamenta.

January 11th, 1826.—All but well.—27th. Dismissed cured.

Case XI.—December 28th, 1825.—Niel M'Vicar, age thirty-five. Conjunctivitis catarrhalis, since the end of October. Has been troubled with this complaint for six preceding winters.

Gtt. Solut. Nitrat. Argent.—Ung. Præcip. Rubr. o. n.

30th.—Symptoms abated.

Collyrium Muriat. Hydrargyri.

January 4th, 1826.—Much improved.

6th.—Eyes more inflamed. Conjunctiva of the lower eyelids somewhat granular.

Applicetur Sulphas Cupri.

22d.—All but well.—February 8th. Dismissed cured.

Case XII.—December 28th, 1825.—Euphemia Wilson, age twenty-six.—Conjunctivitis catarrhalis of the right eye, of three weeks' standing, attended at first with considerable rheumatic pain of the head and cheek. Eyelids considerably swelled.

Scarif. facies interna palp. inf. dext.—Ung. Præcip. Rubr. o. n.

30th.—Gtt. Solut. Nitrat. Argent.—Collyrium Mur. Hydrarg.

January 2d, 1826.—Much improved.

30th.—Dismissed cured.*

Case XIII.—December 28th, 1825.—Charlotte Wilson, age one year. Conjunctivitis catarrhalis, of a fortnight's standing. Eyelids much swelled, and a profuse puriform discharge. Has probably received the disease from her mother, the subject of Case XII.

Injiciat. Solutio Sulphatis Cupri.—Ung. Præcip. Rubr. o. n.

30th.—Eyelids somewhat fallen, and puriform discharge rather less.

Collyrium muriat. hydrarg.

January 2d, 1826.—Considerably improved.

Gtt. Solut. Nitrat. Argent.—Cont. Collyrium et Unguentum.

4th.—Still a considerable discharge of puriform matter, with much swelling of the palpebral conjunctiva.

Injiciat. Solutio Sulphatis Cupri.

9th.—Swelling somewhat fallen.

Gtt. Solut. Nitrat. Argent.

16th.—Much improved.—30th. Dismissed cured.

Case XIV.—January 4th, 1826.—Rebecca Chattie, age one year. Conjunctivitis puro-mucosa, of ten days' standing. Has probably received the disease from her brother.

Gtt. Solut. Nitrat. Argent.—Collyrium Muriat. Hydrarg.

6th.—Puriform discharge diminished.

* This case, though one of catarrho-rheumatic ophthalmia, I introduce here in connexion with Case XIII.

11th.—Puriform discharge ceased.

13th.—Ung. Præcip. Rubr. o. n.

16th.—Dismissed cured.

Case XV.—January 11th, 1826.—Hugh M'Gregor, age fifty-nine. Conjunctivitis catarrhalis, of a month's standing. Symptoms subsiding. Complaint began in the right eye, but now affects the left chiefly.

Gtt. Solut. Nitr. Argent.—Sulphat. Magnes. ℥ jss.—Ung. Præc. Rub. o.n.

13th.—Collyrium Muriat. Hydrarg.—Pilulæ Aloet. i. mane et vespere.

25th.—Eyes all but well.

Case XVI.—January 11th, 1826.—William Leck, age fifty-six. Conjunctivitis catarrhalis; frequently, during the last five or six years, Distichiasis.

Evulsio pseudo-ciliorum.—Gtt. Solut. Nitrat. Argent.—Ung. Præcip. Rubr. o. n.

13th.—Eyes much relieved.

Collyrium Muriat. Hydrar.—Cont. Unguentum et Solutio.

February 1st.—Dismissed cured.

Case XVII.—January 23d, 1826.—John Taylor, age thirty-six. Severe conjunctivitis puro-mucosa, affecting particularly the right eye, the conjunctiva of which is considerably chemosed, and the cornea hazy. A feeling of sand in the eyes, but no headache. Bowels open. A blister on the nape of the neck, and another behind the right ear, discharging.

Scarif. facies interna palp. inf. dext.—Gtt. Solut. Nitr. Argent.—Collyr. Muriat. Hydr.—Ung. Præcip. Rubr. o. n.—Pediluvium tepidum h. s.

25th.—Chemosis of the right eye considerably less; feeling of sand in the eyes, and all the other symptoms, abated.

Cont. remedia.

30th.—Chemosis gone; no discharge from the eyes.

Cont. Solutio, Collyrium, et Unguentum.

February 1st.—Eyes nearly well.—6th. Dismissed cured.

Case XVIII.—January 27th, 1826.—John Newlands, age six. Conjunctivitis catarrhalis of both eyes, of twelve days' standing. A feeling of sand in the eyes.

Gtt. Solut. Nitr. Argent.—Ung. Præcip. Rub. o. n.—Collyr. Mur. Hydr.—Subm. Hydr. gr. iij.; Sacch. Alb. gr. vj. M. capiat q. p.

30th.—Symptoms much abated.

Cont. Solutio, Collyrium, et Unguentum.

February 10th.—Dismissed cured.

Case XIX.—May 5th, 1826.—Elizabeth Balfour, age twenty-one. Conjunctivitis catarrhalis, of three days' standing, affecting the right eye. Has been subject to this complaint for a twelvemonth past; but it has never affected the left eye.

Sulph. Magnes. ℥ jss.—Gtt. Solut. Nitr. Argent.—Ung. Præc. Rub. o. n.

8th.—Symptoms considerably better.

Collyrium Muriat. Hydrarg.

June 5th.—Eye perfectly well. Dismissed cured.

Case XX.—May 10th, 1826.—Agnes Donald, age three months. Conjunctivitis catarrhalis since the 7th, affecting both eyes.

Scarif. facies interna palpebr. infer.—Gtt. Solut. Nitrat. Argent.—Ung. Præcip. Rub. o. n.—Collyrium Muriat. Hydrarg.

12th.—Much improved.

Cont. medicamenta.

15th.—All but well.—22d. Dismissed cured.

see vol. lvii. p. 37.

*Spreull's-court, Glasgow; 22d June,* 1826.

---

